# A portable EEG signal acquisition system and a limited-electrode channel classification network for SSVEP

**DOI:** 10.3389/fnbot.2024.1502560

**Published:** 2025-01-15

**Authors:** Yunxiao Ma, Jinming Huang, Chuan Liu, Meiyu Shi

**Affiliations:** College of Engineering, Qufu Normal University, Rizhao, China

**Keywords:** Brain-Computer Interfaces (BCIs), Robot Operating System (ROS), BCI systems, steady-state visual evoked potential (SSVEP), limited channels

## Abstract

Brain-computer interfaces (BCIs) have garnered significant research attention, yet their complexity has hindered widespread adoption in daily life. Most current electroencephalography (EEG) systems rely on wet electrodes and numerous electrodes to enhance signal quality, making them impractical for everyday use. Portable and wearable devices offer a promising solution, but the limited number of electrodes in specific regions can lead to missing channels and reduced BCI performance. To overcome these challenges and enable better integration of BCI systems with external devices, this study developed an EEG signal acquisition platform (Gaitech BCI) based on the Robot Operating System (ROS) using a 10-channel dry electrode EEG device. Additionally, a multi-scale channel attention selection network based on the Squeeze-and-Excitation (SE) module (SEMSCS) is proposed to improve the classification performance of portable BCI devices with limited channels. Steady-state visual evoked potential (SSVEP) data were collected using the developed BCI system to evaluate both the system and network performance. Offline data from ten subjects were analyzed using within-subject and cross-subject experiments, along with ablation studies. The results demonstrated that the SEMSCS model achieved better classification performance than the comparative reference model, even with a limited number of channels. Additionally, the implementation of online experiments offers a rational solution for controlling external devices via BCI.

## 1 Introduction

Brain-Computer Interfaces (BCIs) are a rapidly evolving field situated at the convergence of neuroscience, signal processing, and artificial intelligence, providing transformative opportunities for human-computer interaction. BCIs capture brain signals through invasive or non-invasive devices and translate an individual's intentions into commands that control external systems, enabling direct communication between the brain and these systems (Arpaia et al., 2020; Li et al., 2023). This technology holds significant promise not only for patients with neurological disorders but also for individuals with motor impairments, offering them the ability to control external devices, such as prosthetics, wheelchairs, and robots, thereby enhancing their autonomy and quality of life (Zhang et al., 2023; Lu et al., 2020). Beyond assistive technologies, BCIs are also being explored in various other fields, including entertainment, communication, and neurorehabilitation, where they can facilitate tasks ranging from controlling video games and smart home devices to aiding in motor recovery post-stroke (Wu et al., 2022; Yao et al., 2020). In addition, with the rapid development of intelligent driving technology, BCIs is increasingly being applied within this field. Wang et al. (2018) and Xu et al. (2021) effectively utilized BCI technology to explore its potential applications in driving scenarios. Through the integration of BCI technology with advanced algorithmic models, they achieved precise monitoring and real-time alerting of driver fatigue.

Despite the substantial potential demonstrated by EEG-based Brain-Computer Interface (BCI) systems, their transition to everyday applications remains constrained by several factors. For instance, signal acquisition is typically conducted in low-interference laboratory environments, which, while ensuring high-quality data collection, are not practical for maintaining a consistently low-noise standard in real-world settings (Al-Fahoum and Al-Fraihat, 2014; Minguillon et al., 2017; Valentin et al., 2018). Additionally, many EEG-based BCI systems rely on numerous wet electrodes to capture the necessary brain signals. This method involves the use of conductive gel to maintain proper contact with the scalp, which, although it enhances signal quality, is complex, time-consuming, and impractical for everyday use (Xing et al., 2018; Wang et al., 2021; Mhapankar and Shah, 2022). Furthermore, the dependence on a large number of electrodes not only increases the complexity of the setup but also introduces challenges in signal processing. These limitations underscore the difficulties in transitioning BCI technology from experimental research to practical, user-friendly applications in daily life (Tam et al., 2011; Ramirez-Quintana et al., 2021).

To address the challenges associated with traditional EEG-based Brain-Computer Interface (BCI) systems, portable and wearable EEG devices have emerged as promising solutions. These devices offer significant advantages in terms of user convenience and practicality (He et al., 2023). For example, dry electrode systems eliminate the need for conductive gel, simplifying the setup process and enhancing user comfort (Li et al., 2020). Wang et al. skillfully employed a wireless dry-electrode EEG acquisition system (model HD-72, manufactured by Cognionics Inc., USA) to collect extensive EEG data from a large number of participants, establishing a robust dataset for their comprehensive studies in this domain. This data collection effort laid a solid foundation for their in-depth research into various aspects of EEG-based fatigue detection and cognitive state analysis, as demonstrated in their subsequent works (Wang et al., 2020a,c,b; Chen C. et al., 2023). Portable EEG devices, such as those developed by Emotiv EPOC+ (Chabin et al., 2020) and Muse headband (Krigolson et al., 2021), are designed to be lightweight and easy to use, making them suitable for real-world applications. These advancements have paved the way for more accessible and user-friendly BCI systems. However, despite their benefits, wearable EEG devices face limitations due to the constrained number of electrodes, which can lead to a decline in BCI performance (Li et al., 2024). Several studies have examined the impact of using a limited number of electrodes or non-feature region channels for intent classification in BCIs. Lan et al. (2021) demonstrated that combining non-occipital signals and reference points could yield an accuracy of around 80%. Hsu et al. (2015) showed that SSVEP signals from the frontal region could effectively support BCIs. In scenarios with limited occipital channels, Ge et al. (2021) achieved 76% accuracy with just three occipital channels. Likewise, Chen et al. (2017) obtained accuracies of 86.58% in simulations and 85.54% in real-world robot control using a single channel. These results suggest that non-feature region electrodes can be leveraged for intent classification when necessary.

Additionally, while mainstream BCI acquisition and analysis platforms, such as OpenBCI, EEGLab, and OpenVibe, perform well in EEG signal acquisition and processing, they lack integrated solutions for controlling external devices (Tonin et al., 2022; Beraldo et al., 2018). In recent years, several approaches have been proposed to drive external devices using the aforementioned BCI systems. For example, Casey et al. (2021) developed a BCI-based robotic arm control solution using the OpenBCI platform to assist in the rehabilitation of patients with neurological impairments. Similarly, An et al. (2024) introduced a robot control system based on OpenViBE to enhance the practicality of BCI applications. Shao et al. (2020) utilized the EEGLab toolbox to study and effectively control a wall-climbing cleaning robot. While these studies present control solutions for external devices based on specific BCI systems, their implementation requires developing customized underlying code for each external device, which limits their integration efficiency. However, despite these advancements, these platforms primarily focus on signal processing capabilities and still require significant development to achieve seamless integration with real-world applications, particularly in complex environments and robotic interactions. With the advancement of robotic technologies, the Robot Operating System (ROS) has become a widely adopted development tool (Quigley et al., 2009; Lee, 2021). Known for its high integrability with external devices, ROS provides a robust framework for seamlessly integrating diverse hardware and software components in robotics and automation. It facilitates efficient communication between sensors, actuators, and computational nodes, making it an ideal choice for controlling complex systems (Caldas et al., 2024).

To address the aforementioned issues, the main contributions of this study are as follows:

i. A ROS-based framework, Gaitech BCI, is proposed for BCI signal acquisition and analysis using the portable dry-electrode device H10C. The platform can be used to manage EEG experiments, create datasets, and export data in formats such as .mat, .fif, and .csv through a user interface;
ii. Three ROS packages were developed and integrated with the BCI system, providing a solution for controlling external devices through the BCI system;
iii. To address the issue of reduced BCI classification performance caused by the limited number of channels in portable EEG devices, a novel channel selection network based on SE attention and multi-scale convolution is proposed, which extracts both feature and non-feature channel information;
iv. The framework and network are validated through SSVEP data collection from ten subjects, including within-subject, cross-subject, and ablation experiments, as well as real-time testing, demonstrating their effectiveness.

The rest of this article is organized as follows: Section 2 describes the proposed system framework, classification model framework, and acquisition of experimental data, Section 3 describes experimental details and result analysis, and Section 4 summarizes our work and discusses it. The model code and 3 python packages can be obtained at https://github.com/chiwuan6/SEMSCS.

## 2 Materials and methods

**Figure 1:**
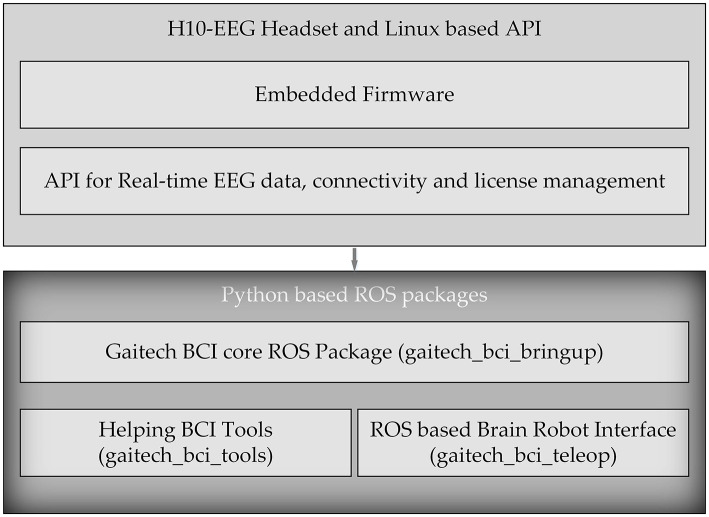
Overall architecture of Gaitech BCI.

In this study, we developed an electroencephalography (EEG) acquisition and analysis system named Gaitech BCI using the H10C dry electrode portable BCI device. The design of this platform enables users to intuitively configure EEG-based BCI experiments and create datasets for both offline analysis and online experiments. The overall architecture of Gaitech BCI is illustrated in Figure 1. The Gaitech BCI system is composed of two main components: the upper layer, comprising the H10-EEG Headset and a Linux-based API, and the lower layer, consisting of Python-based ROS packages. The upper layer includes two key elements: the embedded firmware that drives the hardware device and the API interface responsible for device connectivity and data transmission. The lower layer consists of three custom-developed ROS packages. Raw signals are initially acquired by the hardware device, driven by the embedded firmware in the upper layer, and then transmitted via the API interface to the ROS environment in the lower layer. Within the ROS environment, these signals are exchanged among the ROS packages through topics and services, enabling seamless integration and data flow. The three packages in the lower layer will be described in detail later, and the following is an overview of the three functional packages:

- gaitech_bci_bringup: As the core package of the framework, this package contains all the necessary services and topics for managing the device and acquiring raw EEG data from it;
- gaitech_bci_tools: This package serves as the system's tool management package, integrating various functionalities. Through ROS nodes, users can invoke corresponding tools to assist with experiments. Additionally, it includes a graphical user interface (GUI) that provides access to the required functions.
- gaitech_bci_teleop: This package acts as the interface between the system and external devices, allowing users to configure experiments for online or simulation validation. Users can publish image topics in ROS to receive visualized images or interfaces as needed.

### 2.1 Device

**Figure 2:**
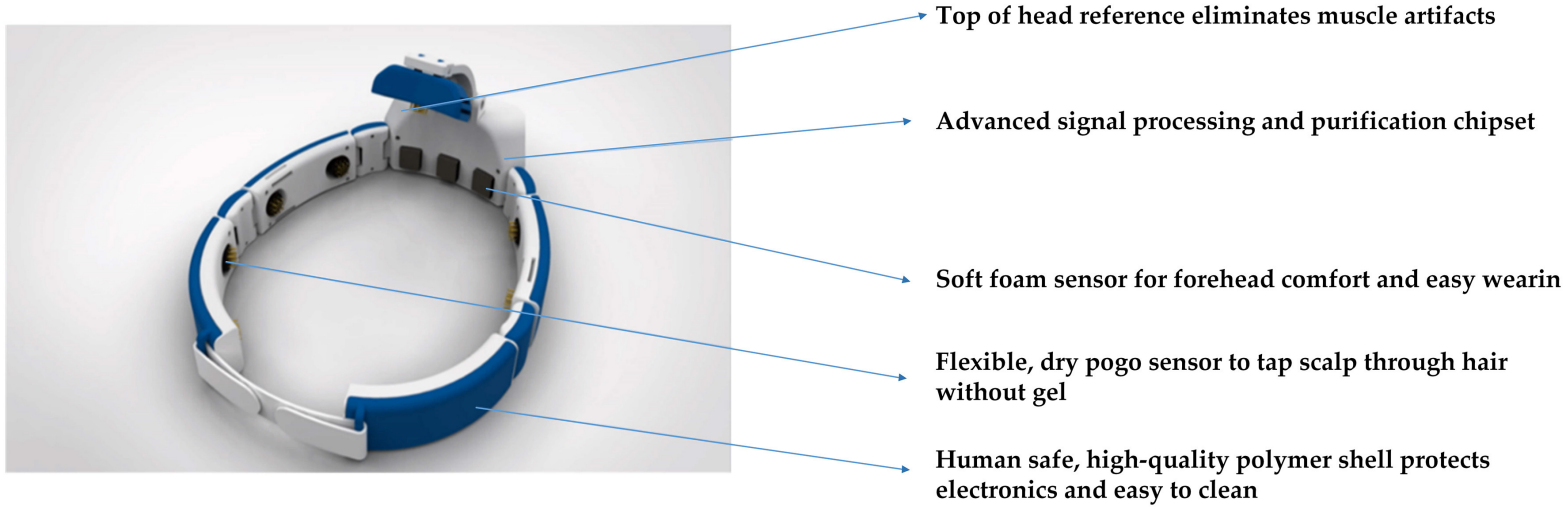
Several characteristics of the Avertus H10C EEG Headset.

The signal acquisition device used in the proposed Gaitech BCI system is the Avertus H10C EEG headset. The H10C has been reasonably designed for functionality and comfort. Its lightweight, ergonomic design allows for multiple hours of comfortable use. Foam electrodes are used on FP1, FP2, and FPz to provide comfort where there is no hair. Compared to traditional wet electrodes, foam electrodes eliminate the need for conductive gel, reducing preparation time and the inconvenience associated with cleaning (Liao et al., 2012; Searle and Kirkup, 2000). Unlike dry electrodes, which may suffer from inconsistent signal quality due to varying scalp impedance, foam electrodes offer a balance of comfort and reliable signal acquisition, especially in areas without hair (Chi et al., 2011). However, foam electrodes may not provide the same signal accuracy as wet electrodes in certain high-precision applications (Yeung et al., 2015). Spring-loaded, gold-plated electrodes at FCz, F7, F8, T3, T4, T5, T6, O1, and O2 are designed for hair penetration, ensuring comfort and delivering high-quality EEG signals. Both the foam and spring-loaded electrodes are removable for easy cleaning and replacement when needed. The H10C is adjustable with easy-to-use Velcro straps because of its ability to conform to most head shapes and sizes. The characteristics of H10C are shown in Figure 2.

### 2.2 Core packages

#### 2.2.1 gaitech_bci_bringup

**Figure 3:**
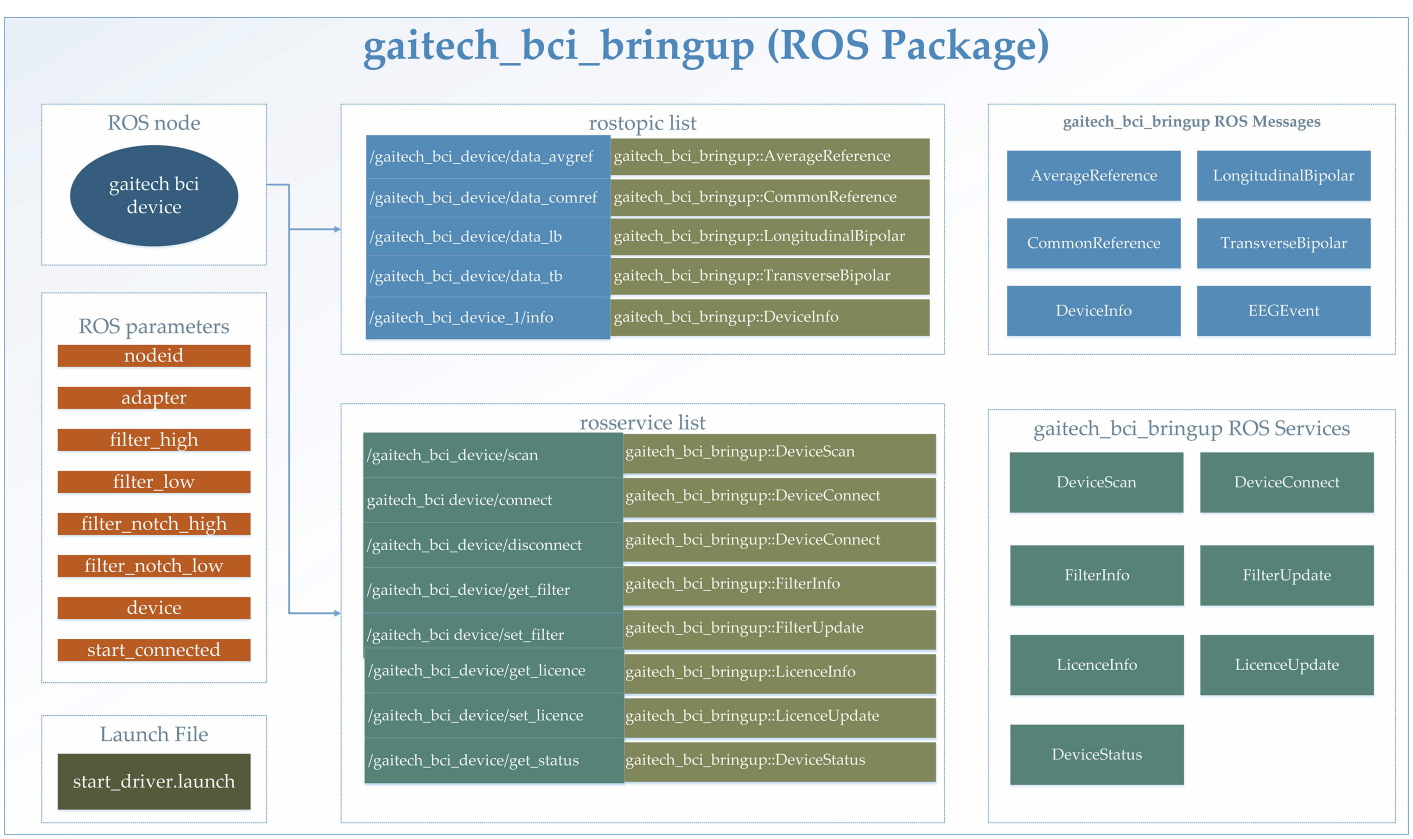
Detailed description of the gaitech_bci_bringup ROS package.

In this study, the gaitech_bci_bringup ROS package was utilized for the comprehensive processing of BCI data. Specifically designed for BCI applications, this package encapsulates a complete workflow for data acquisition, preprocessing, and state monitoring, facilitating seamless integration into ROS-based research environments. A detailed explanation of the package is provided in Figure 3. An itemized description of the functionalities included in this package is provided in the following:

- Data acquisition: The gaitech_bci_bringup package establishes a communication interface with BCI devices, enabling the subscription and aggregation of raw data streams, including EEG signals and device status information. This feature ensures the timely capture of BCI data. The support for different devices can also be expanded by users based on the communication methods of their devices;
- Data preprocessing: By incorporating classical filtering algorithms, such as high-pass, low-pass, and notch filters, gaitech_bci_bringup allows researchers to tailor the data to their specific analysis requirements. Additionally, it supports the conversion of raw data into various reference formats (e.g., Average Reference, Common Reference, Longitudinal Bipolar, and Transverse Bipolar) (Acharya and Acharya, 2019);
- State monitoring and services: The package incorporates a state monitoring system, which continuously tracks the status of BCI devices and filters. Through ROS services and topics, gaitech_bci_bringup provides researchers with real-time access to device connectivity status, filter configurations, and other pertinent information (Koubâa et al., 2017);
- Configuration and deployment: To facilitate ease of use and reproducibility, allowing researchers to configure the package to their specific needs. Furthermore, the inclusion of launch files simplifies the deployment process (Koubâa et al., 2017), enabling researchers to quickly bring up the entire BCI data processing environment with a single command.

#### 2.2.2 gaitech_bci_tools

**Figure 4:**
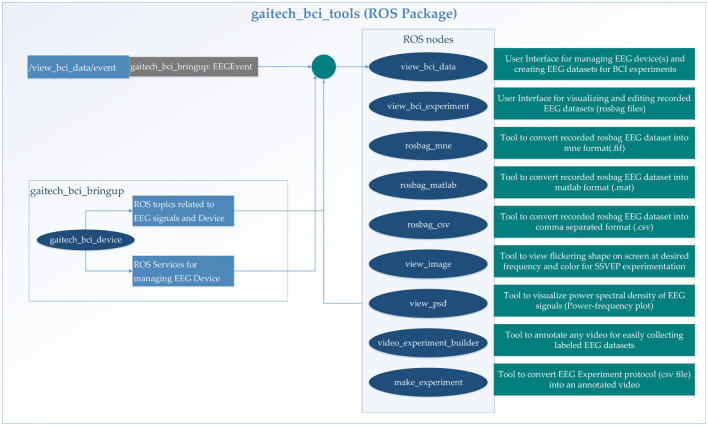
Detailed description of gaitech_bci_tools.

The gaitech_bci_tools package facilitates EEG experiments and analysis within a ROS framework, providing tools for real-time monitoring, data processing, and device management. These functionalities, along with integration with gaitech_bci_bringup for device setup, are accessible through a graphical user interface (GUI). The structural overview of the package is presented in Figure 4, with specific details outlined as follows:

- User interface: The gaitech_bci_tools package provides a user interface for managing EEG devices. Users can view bio data and bio events in real-time, facilitating monitoring and debugging during experiments. Additionally, the package includes a view_bci_data tool, which creates EEG datasets for BCI experiments, allowing researchers to access and analyze recorded data;
- Data conversion tools: A key feature of the gaitech_bci_tools package is its suite of data processing and conversion tools. These tools enable researchers to convert recorded EEG datasets from ROS bag files into various formats, including MNE (.fif), MATLAB (.mat), and CSV (.csv), thereby enhancing the interoperability of EEG data with other analysis software;
- ROS services for EEG device management: The package incorporates ROS services that facilitate the management of EEG devices (Koubâa et al., 2017). For instance, the view_psd service enables researchers to visualize the power spectral density of EEG signals, providing insights into the frequency content of the recorded data. Additionally, the video_experiment_builder tool simplifies the process of annotating videos and collecting labeled EEG datasets, while the make_experiment service converts EEG experiment protocols (in CSV format) into executable experiment configurations;
- Integration with gaitech_bci_bringup: The gaitech_bci_tools package seamlessly integrates with the gaitech_bci_bringup ROS package, which handles the initial setup and configuration of EEG devices. This integration ensures that researchers can quickly bring up their EEG data processing environment, from device connection to data visualization and analysis, within a unified ROS-based framework.

#### 2.2.3 gaitech_bci_teleop

**Figure 5:**
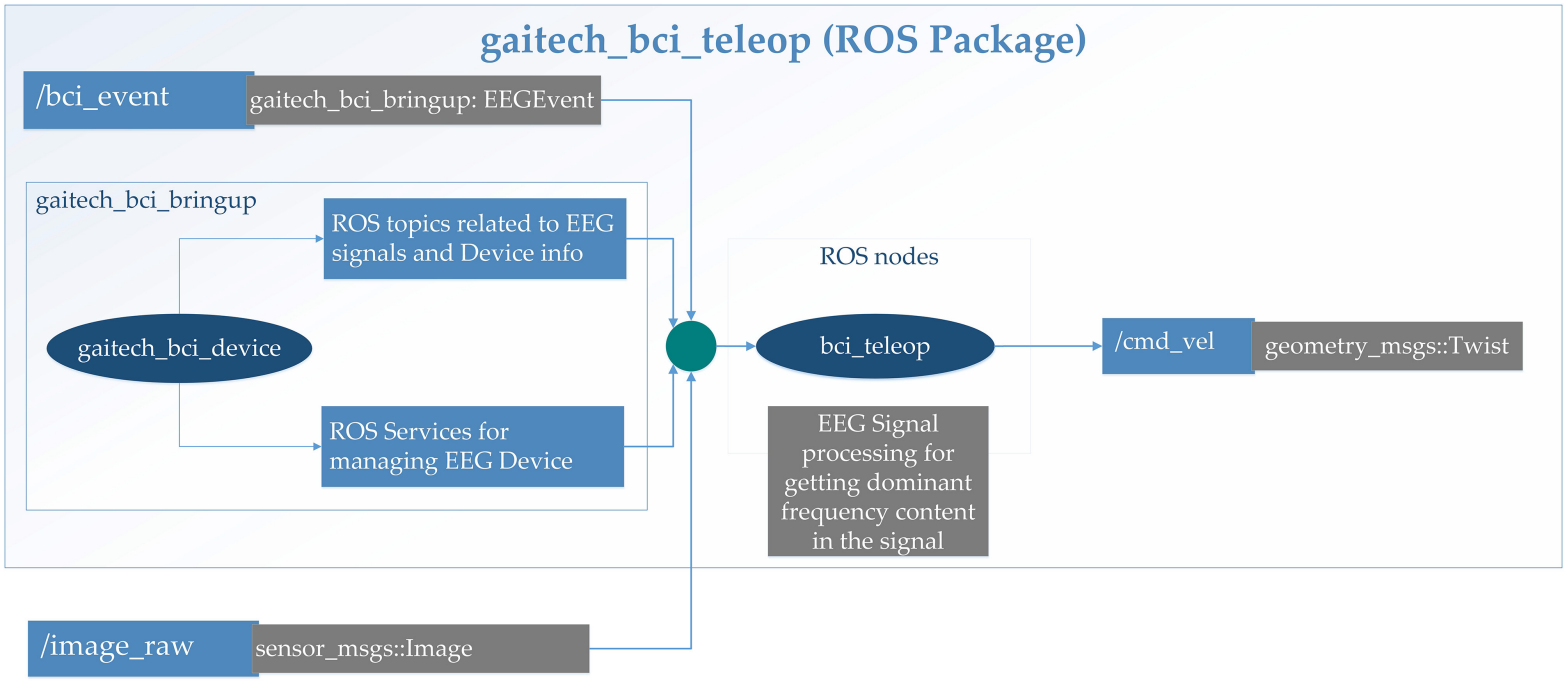
Detailed description of gaitech_bci_teleop ROS package.

This package serves as a communication interface between the system and external devices, leveraging the ROS capability of cross-language and cross-version communication, enabling the flexible deployment of signal recognition algorithms and establishing a bridge for interactions between the system and external peripherals (Koubâa et al., 2017; Fairchild and Harman, 2016). The structural organization of the package is illustrated in Figure 5, and its main features are as follows:

- EEG signal transmission: The EEG signals are captured through the gaitech_bci_device hardware interface, and these signals along with device information are transmitted within the ROS network in the form of ROS topics. This approach facilitates seamless data flow and integration with the ROS ecosystem (Koubâa et al., 2017; Fairchild and Harman, 2016);
- Robot control command generation: Based on the analytical outcomes of the EEG signals, corresponding robot control commands are generated and dispatched to the robot via ROS standard topics (e.g., /cmd_vel) (Koubâa et al., 2017; Fairchild and Harman, 2016), thereby directing its movements. This mechanism allows for dynamic and responsive control strategies based on brain-computer interaction;
- ROS interface design: Through the modular architecture of ROS nodes and services, this package offers interfaces that simplify integration and extension with other ROS packages or system components (Koubâa et al., 2017; Fairchild and Harman, 2016).

Overall, the interaction and data flow between the three core ROS packages are as follows. The data flow begins with signals transmitted from upper level BCI devices, which are received and processed by gaitech_bci_bringup. This package is responsible for raw EEG signal acquisition and preprocessing, including filtering and formatting the data for subsequent analysis.

The preprocessed data is then passed to gaitech_bci_tools, which offers a graphical interface for real-time monitoring, dataset creation, and visualization. This interface also enables users to interactively modify the preprocessing parameters of gaitech_bci_bringup, facilitating system customization and adaptability to specific experimental requirements.

**Figure 6:**
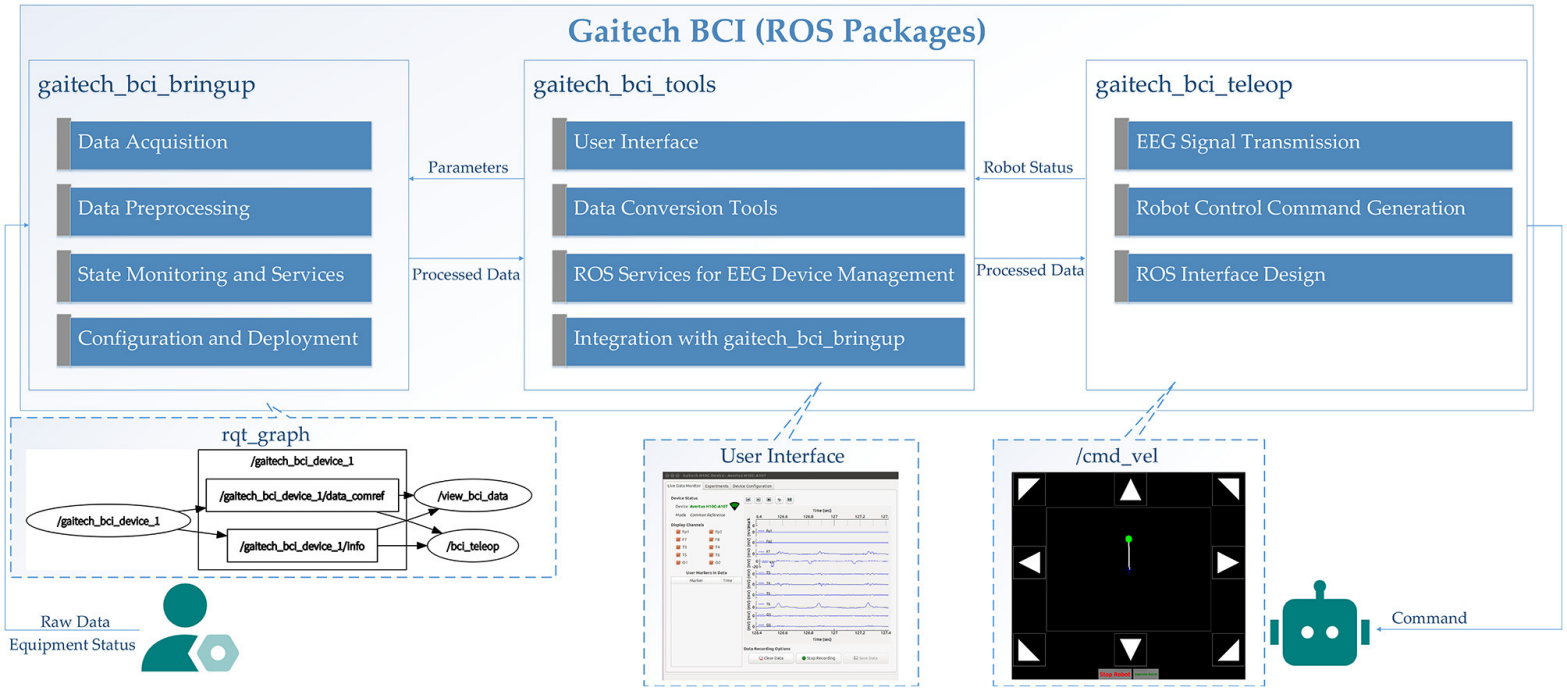
Data flow and interaction between core ROS packages in the Gaitech BCI framework.

Finally, the processed datasets and outputs from gaitech_bci_tools are utilized by gaitech_bci_teleop to generate robot control commands. These commands, based on EEG signal interpretations, are transmitted through the ROS network to execute robot actions. This hierarchical data flow and modular integration establish a pipeline from signal acquisition to real-world application, with each package contributing a distinct but interconnected layer of functionality, thereby supporting a ROS-based BCI research framework. The above description is shown in Figure 6.

### 2.3 Network structure

**Figure 7:**
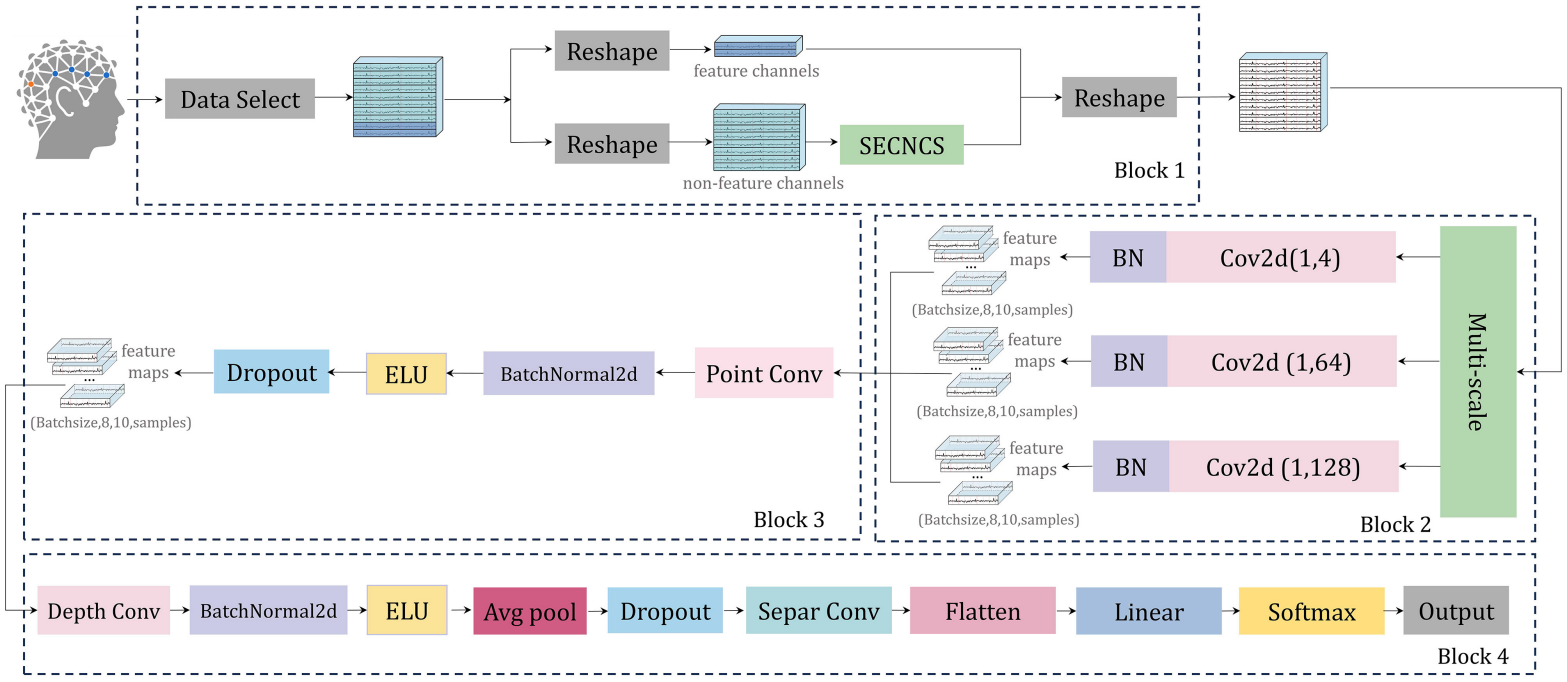
The architecture of SEMSCS, consisting of four main blocks: (1) an improved SE attention mechanism (Block 1), (2) a multi-scale convolutional layer (Block 2), (3) a pointwise convolutional layer (Block 3), and (4) a block combining deep convolutional and separable convolutional layers (Block 4).

In this research, we introduce a neural network architecture called SEMSCS, which integrates the SE attention mechanism (Hu et al., 2018; Li et al., 2021) with multi-scale convolution (Ko et al., 2021; Liu et al., 2022). This architecture leverages both channel attention and multi-scale convolution techniques to extract features from the complete set of channels with limited electrode data. The SEMSCS model is depicted in Figure 7. The design draws inspiration from EEGNet (Lawhern et al., 2018), incorporating elements of channel attention and multi-scale convolution for improved feature extraction.

The SEMSCS model is composed of four key components: a channel selection and attention block based on an improved SE block, a multi-scale convolution block, a pointwise convolution block, and a final processing layer consisting of depthwise and separable convolutions. Initially, the raw EEG data is reshaped into two parts: paradigm-related feature channel data and non-feature channel data. This study employs the SSVEP paradigm to experimentally evaluate the network and system. The feature channels are electrodes located in the occipital region. For the dry electrode device H10C, which contains 10 channels, the data is divided into two parts accordingly: the non-feature channels correspond to the 8 electrodes in non-occipital regions, while the feature channels correspond to the 2 electrodes in the occipital region (O1, O2). This configuration was selected to align with the experimental paradigm in this study and to adapt to the electrode layout of the device used. Importantly, the proposed network is adaptable and allows flexible feature channel selection during initialization, accommodating different devices and electrode distributions based on specific experimental requirements. Next, latent features from the non-feature channels are extracted using the improved SE block (SECNCS).

**Figure 8:**
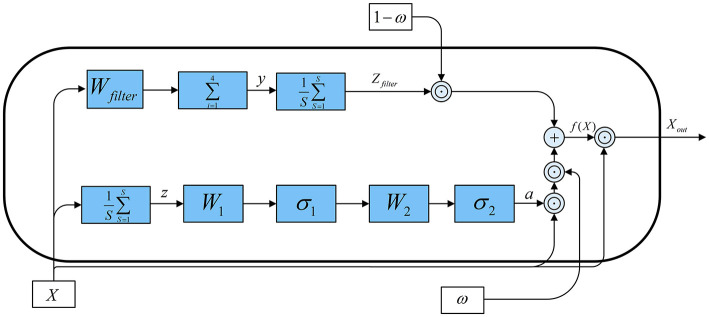
The architecture of the improved SE block (SECNCS).

The structural overview of the SECNCS is presented in Figure 8. The input data for SECNCS is denoted as *X*, *X* ∈ ℝ^*B*×*C*×*S*^, where *B* is the batch size, *C* is the number of channels, and *S* is the length of the time series. Then, *X* flows into two branches. In the first branche, the global information of each channel is obtained primarily by aggregating data across the time dimension. This process can be formulated as $z=\frac{1}{s}\overset{s}{\underset{s=1}{\sum }}X_{c,s}$. Subsequently, two linear transformations are applied. The former transformation, with weight matrix $W_{1}\in {\mathbb{R}}^{C\times C/r}$, compresses thechannel dimension. Meanwhile, a nonlinear activation function σ_1_ (relu) is adopted to enhance the model's attention for the important features. Further, the later linear transformation with $W_{2}\in {\mathbb{R}}^{C/r\times C}$, restores the original channel dimension after applying activation function σ_2_ (sigmoid). This process generates the attention weights *a*, which reflect the importance of each channel. The attention weights are then multiplied with the input data channel-wise to produce the output features *X*_1_ of the first branch. In the second branch, channel data are processed along the time dimension using local weighted summation, similar to a convolution operation. Specifically, a filter weight vector *W*_filter_ is applied to compute the weighted sum across segments of the time series, producing the filtered data *y*. This filtered result is then aggregated along the time dimension and acts as a one-dimensional mean pooling operation that enhances the contrast of feature information to obtain the features *Z*_*filter*_ of the second branch. In the end, a learnable weight vector ω ∈ ℝ^*C*^ is introduced to combine the results from the two branches. We stress that value of ω is constrained between 0 and 1 by using the sigmoid function. This weight controls the combination ratio of the features from the two branches. Consequently, the fusion feature weight can be obtained as:


(1) $$\begin{array}{l} f\left(X\right)=\omega ⊙X_{1}+\left(1-\omega \right)⊙Z_{filter} \end{array}$$

where: ⊙ represents element-wise (Hadamard) multiplication, *a* = σ_2_(*W*_2_σ_1_(*W*_1_*z*)), *X*_1_ = *a* ⊙ *X*, $Z_{filter}=\frac{1}{S}\overset{S}{\underset{i=1}{\sum }}y_{c,s}$, $y_{c,s}=\overset{4}{\underset{i=1}{\sum }}W_{\text{filter},i}X_{c,i:i+s-1}$ and in this study *r* = 8.

Finally, the fused features are multiplied element-wise with the original input data to produce the final output *X*_out_, given by:


(2) $$\begin{array}{l} X_{\text{out}}=f\left(X\right)⊙X \end{array}$$

The processed non-feature channel data, along with the original feature channel data, are merged along the channel dimension to restore the original data shape before being fed into the multi-scale convolution layer. This layer contains three parallel convolution operations: one with a kernel size of (1, 4) to capture short-range dependencies, another with a kernel size of (1, 64) to extract mid-range dependencies, and a third with a kernel size of (1, 128) to capture long-range dependencies (Tao et al., 2023). Batch normalization is applied after each convolution to ensure a stable learning process. The outputs from these convolutions are concatenated along the feature map dimension to create a unified feature map.

Subsequently, the unified feature map is processed through a pointwise convolution layer, employing a 1 × 1 convolution to integrate the three sets of feature maps from the previous multi-scale layer into one. This step reduces the dimensionality of the combined feature maps to match the number of input channels (Ko et al., 2021), followed by batch normalization and ELU activation, which contribute to maintaining network stability and effective regularization.

The final layers consist of a depthwise convolution, where a channel-wise operation is performed, followed by batch normalization, ELU activation, average pooling, and dropout. The separable convolution layer further refines the feature map through depthwise and pointwise convolutions, again followed by batch normalization, ELU activation, average pooling, and dropout. Ultimately, the processed feature map is flattened and passed through a fully connected layer for classification, with the output probabilities calculated via a softmax function (Lawhern et al., 2018).

### 2.4 Signal acquisition

**Figure 9:**
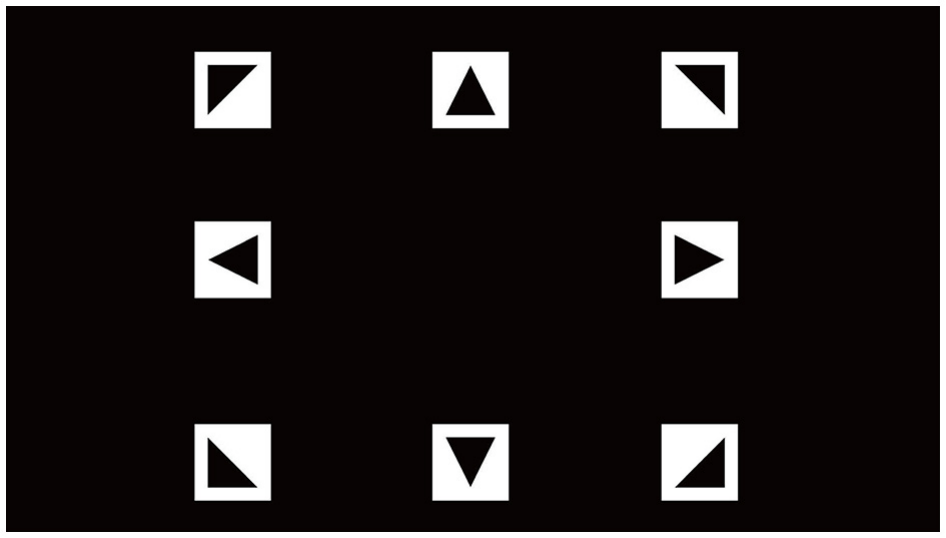
SSVEP experiment stimulation interface.

In this study, we conducted an SSVEP-based experiment using a custom-designed Gaitech BCI system to record EEG signals. Visual stimuli were presented on a 21.5-inch LCD monitor with a resolution of 1,920 × 1,080 pixels and a refresh rate of 60 Hz. Eight visual targets, arranged in a 3 × 3 matrix with the center target removed, were displayed to participants. To ensure precise modulation of the flickering stimuli, we employed a joint frequency-phase modulation (JFPM) method to control the sinusoidal flicker of each target. This approach allowed for accurate manipulation of both frequency and phase for the generation of SSVEP (Wang et al., 2016; Pan et al., 2022).


(3) $$\begin{array}{l} C_{i}\left(t\right)=\sin\left(2\pi f_{i}t+\phi_{i}\right) \end{array}$$

where *C*_*i*_(*t*) represents the contrast of the *i*-th target at time *t*, *f*_*i*_ is the flickering frequency of the *i*-th target, and ϕ_*i*_ denotes its phase offset. The target frequencies were set at 7, 8, 9, 10, 11, 12, 13, and 15 Hz, with corresponding phase shifts ϕ_*i*_ distributed from 0 to 7π/4. The stimulus interface is shown in Figure 9. The following formulas represent the frequency and phase of the stimulus square at position (*i, j*) (Li et al., 2024).


(4) $$\begin{array}{l} \{\begin{array}{c} f_{1}\left(i,j\right)=7+3i+j,\;\left(i,j\right)=\left(0,0\right),\left(0,1\right),\left(0,2\right),\left(1,0\right),\left(2,2\right)\\ f_{2}\left(i,j\right)=6+3i+j,\;\left(i,j\right)=\left(1,2\right),\left(2,0\right),\left(2,1\right)\\ \phi_{1}\left(i,j\right)=\frac{\pi }{4}\left(3i+j\right),\;\left(i,j\right)=\left(0,0\right),\left(0,1\right),\left(0,2\right),\left(1,0\right)\\ \phi_{2}\left(i,j\right)=\frac{\pi }{4}\left(3i+j-1\right),\;\left(i,j\right)=\left(1,2\right),\left(2,0\right),\left(2,1\right),\left(2,2\right) \end{array} \end{array}$$

The participants were seated at a fixed distance of 70 cm from the monitor, ensuring optimal visibility without strain. A total of 10 healthy volunteers with normal or corrected-to-normal vision participated in the experiment (4 females and 6 males, aged between 22 and 28 years, with a mean age of 24.1 years) (Wang et al., 2016; Karas et al., 2023). Each participant had prior experience with similar experiments and provided informed consent before participation and the protocol was approved by the Biomedical Ethics Committee of Qufu Normal University (2024-126).

**Figure 10:**
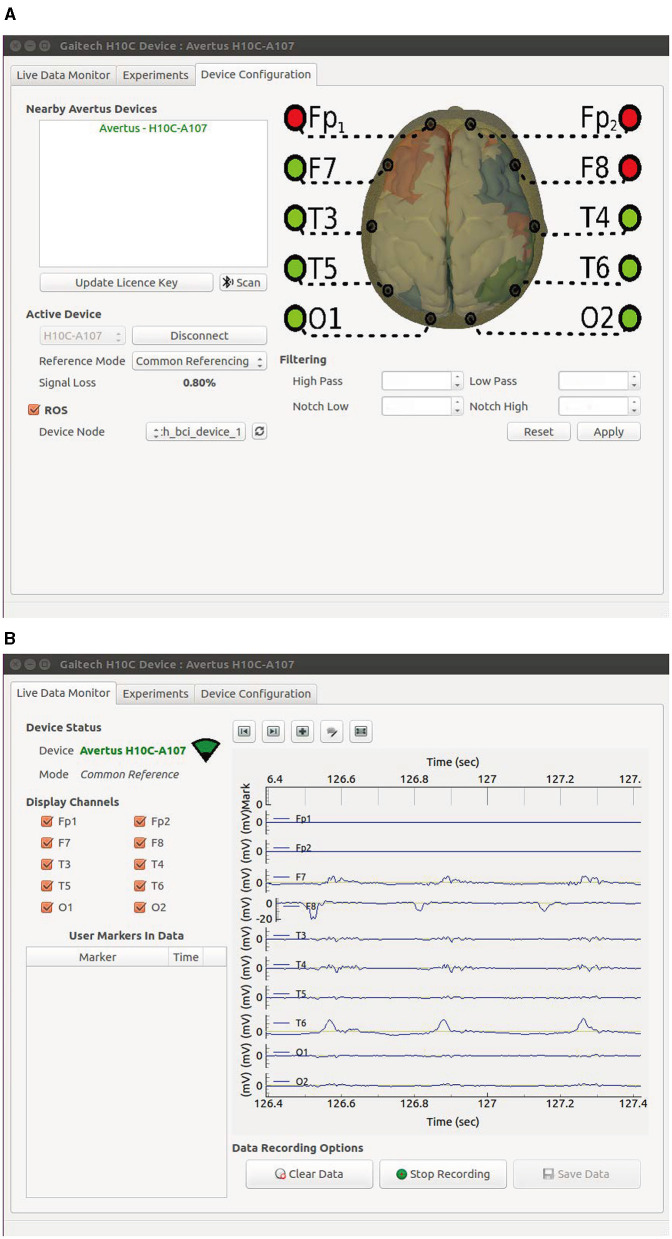
GUI of the Gaitech BCI system. **(A)** Data acquisition parameter setting interface. **(B)** Experiment data preview interface.

During the experiment, participants were required to fixate their gaze on a cued target, indicated by a static highlight for 0.5 seconds before the flickering began. Following the cue, all eight targets flickered simultaneously for 5 seconds, after which the screen was blank for 0.5 seconds before the next trial. Each trial lasted 6 seconds, and participants were instructed to avoid blinking during the 5-second stimulation period. To minimize fatigue, participants were allowed to take breaks between blocks. The sampling frequency was set to 1,000 Hz, and the data were filtered using filters from the gaitech_bci_tools package. The filtering process included a high-pass filter at 6.5 Hz, a notch filter between 47–53 Hz (remove power frequency noise), and a low-pass filter at 65 Hz. After filtering, all data were downsampled to 250 Hz. A total of 40 trials were conducted for each target. The data acquisition interface is presented in Figure 10, with two subfigures included: the parameter setting interface is shown in Figure 10A, while the experiment data preview interface is illustrated in Figure 10B.

In this study, we processed each subject's data through weighted averaging. First, we computed the average of all 40 time series for each frequency file, then aggregated the results across 8 frequencies. Finally, the average of these aggregated results was calculated to obtain the subject's final time series (Wang et al., 2016). This process is represented by the following formula:


(5) $$\begin{array}{l} D_{i}\left(t\right)=\frac{1}{8}\overset{8}{\underset{f=1}{\sum }}\frac{1}{40}\overset{40}{\underset{n=1}{\sum }}d_{i,f,n}\left(t\right) \end{array}$$

where *d*_*i,f,n*_(*t*) denotes the data at time *t* for the *n*-th time series under frequency *f* for subject *i*.

**Figure 11:**
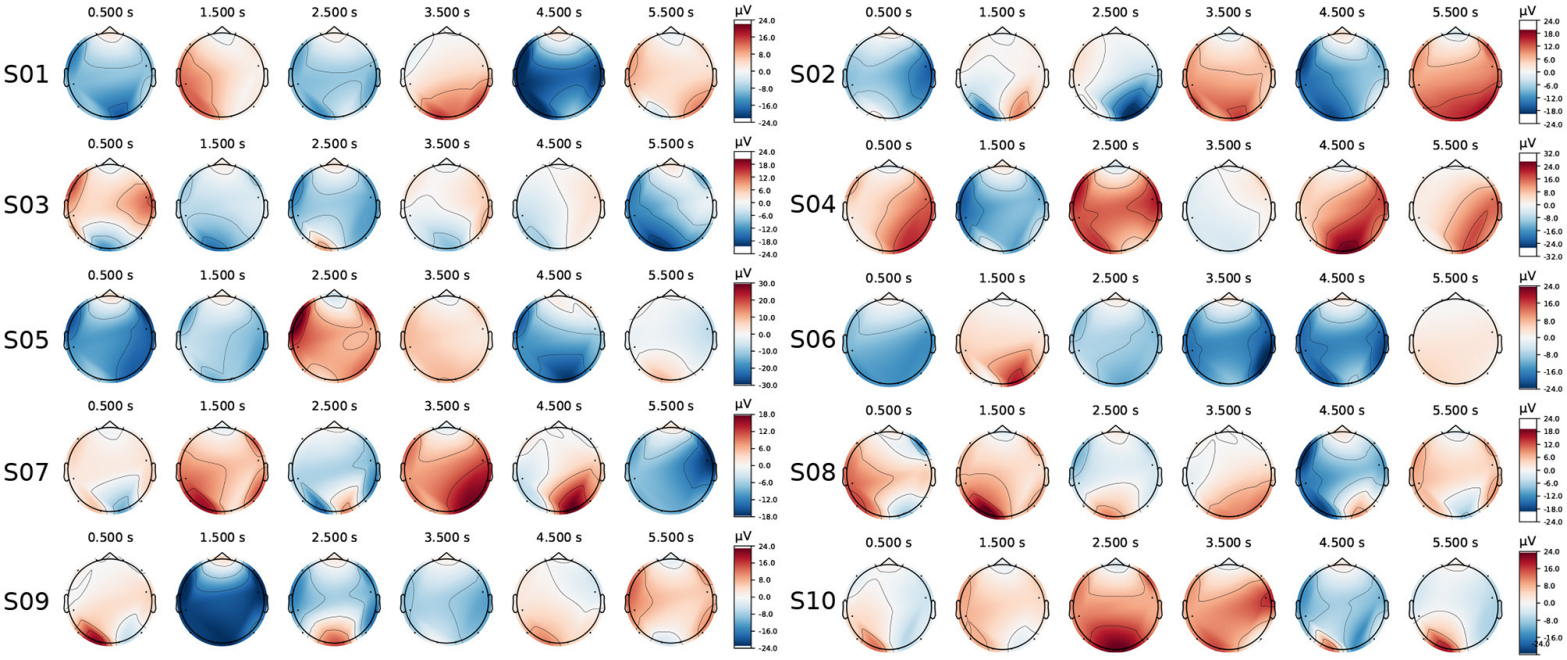
The EEG topographies for each subject (ten subjects in total) were recorded over a 0.5–5.5s interval with a 1s step size.

The EEG topographies for ten subjects were averaged and plotted over a 6-second duration, from 0.5s to 5.5s with a 1s step size, as shown in Figure 11. Notable electrode activity in the occipital region (the feature channel) was observed during stimulation for each subject; however, responses were also evident at other locations (such as the temporal and frontal regions). According to the studies Lan et al. (2021) and Hsu et al. (2015), electrodes in these non-standard feature cortical regions can also provide valuable information for extracting standard features, particularly for portable EEG devices with a limited number of channels.

### 2.5 Decoding methods for comparison

In addition to the SEMSCS, six signal decoding methods were employed in this study, among which Canonical Correlation Analysis (CCA) and Multivariate Synchronization Index (MSI) are classical signal processing and statistical analysis methods. The remaining four methods involved models built and trained using PyTorch.

i) **CCA:** Is a statistical method used to identify and quantify the relationship between two sets of variables by maximizing the correlation between their linear combinations, making it useful for matching EEG signals with stimulus frequencies in SSVEP classification (Lin et al., 2006).
ii) **MSI:** Is a measure used to evaluate the synchronization or phase consistency among multiple EEG signals, which helps in assessing the coherence between brain regions and stimulus frequencies in SSVEP classification (Zhang et al., 2014).
iii) **CNN:** A deep learning model designed for image and signal processing, utilizing convolutional layers to automatically learn spatial hierarchies and features from raw data, which is effective for extracting spatial patterns in EEG signals for classification (Ravi et al., 2020).
iv) **EEGNet:** A compact convolutional network with a single branch that can decode EEG signals of multiple paradigms, consisting of two-dimensional convolution, deep convolution, and separable convolution, has been widely studied (Lawhern et al., 2018).
v) **atten-CCNN:** A convolutional network designed to address the performance degradation of BCI in the case of few channels, integrating attention mechanisms into the convolutional neural network to selectively focus on the most relevant features in EEG signals (Li et al., 2024).
vi) **FB-tCNN:** Incorporating frequency band-specific convolutional layers with transformer blocks, FB-tCNN captures both spectral and temporal features of EEG signals, thereby enhancing the model's performance in SSVEP classification by leveraging frequency-domain information (Ding et al., 2021).

## 3 Experiments and results

### 3.1 Metrics

In this study, all experiments were conducted on a server platform equipped with an Intel(R) Xeon(R) Gold 6240 CPU @2.60GHz and an NVIDIA V100S GPU, using PyTorch 1.12 for model construction. The training of all deep learning models was conducted using five-fold cross-validation. A model checkpoint callback function was applied at the end of each epoch during training to save the model weights with the best classification accuracy, and the saved best model was loaded during the testing phase. Additionally, early stopping was implemented during training to enhance model performance and prevent overfitting, with training halted if no improvement was observed after 50 epochs (Chen J. et al., 2023).

For evaluating the classification performance of the model, both within-subject and cross-subject experiments were conducted. In the within-subject experiments, the average accuracy and Information Transfer Rate (ITR) were used as metrics to assess the model's classification performance. The formula for calculating accuracy is as follows:


(6) $$\begin{array}{l} \text{acc}=\frac{TP+TN}{TP+TN+FP+FN} \end{array}$$

where *TP* is the number of true positives, *TN* is the number of true negatives, *FP* is the number of false positives, and *FN* is the number of false negatives. Accuracy is the proportion of correctly classified samples and is one of the most commonly used evaluation metrics.

The ITR was calculated using the following formula:


(7) $$\begin{array}{l} \text{ITR}=\frac{60}{T}\left(\log_{2}\left(N\right)+P\cdot \log_{2}\left(P\right)+\left(1-P\right)\cdot \log_{2}\left(\frac{1-P}{N-1}\right)\right) \end{array}$$

where *N* is the number of stimulus targets, *P* denotes the classification accuracy, and *T* represents the length of the time window (Yin et al., 2022; Zhang et al., 2024). The ITR is used in within-subject experiments to evaluate the efficiency of the brain-computer interface by quantifying the amount of information transferred per unit time, with higher ITR values indicating better performance in terms of faster and more reliable communication between the brain and the interface.

In cross-subject experiments, the model's performance was evaluated using average accuracy, Kappa value, F1 score, and Precision score. These metrics (excluding average accuracy) are described in detail as follows.

The Kappa statistic measures the agreement between the classification results and random outcomes, with values ranging from –1 (complete disagreement) to 1 (complete agreement). A higher value indicates better consistency. The calculation formula is given by:


(8) $$\begin{array}{l} \kappa =\frac{acc-p_{e}}{1-p_{e}} \end{array}$$

where *acc* represents the observed agreement (or accuracy), and *p*_*e*_ denotes the expected agreement by chance.

The F1 score is the harmonic mean of precision and recall, used to provide a comprehensive evaluation of a model's performance on positive samples. The calculation formula is:


(9) $$\begin{array}{l} F1=2\cdot \frac{\text{Precision}\cdot \text{Recall}}{\text{Precision}+\text{Recall}} \end{array}$$

where Precision is the ratio of correctly predicted positive samples to all samples predicted as positive, and Recall is the ratio of correctly predicted positive samples to all actual positive samples. Their formula is as follows:


(10) $$\begin{array}{l} \text{Precision}=\frac{TP}{TP+FP} \end{array}$$

(11) $$\begin{array}{l} \text{Recall}=\frac{TP}{TP+FN} \end{array}$$

Since Precision, Recall, and Accuracy all rely on the same elements, they provide complementary perspectives on the model's performance. In this study, Recall is included within the F1 score, which combines both Precision and Recall to give a balanced evaluation of the model's ability to classify positive samples. A higher Precision indicates a lower false positive rate, while a higher F1 score suggests a better overall trade-off between Precision and Recall, ensuring both high accuracy in positive predictions and thorough identification of all actual positive samples.

### 3.2 Within-subject experiment

**Figure 12:**
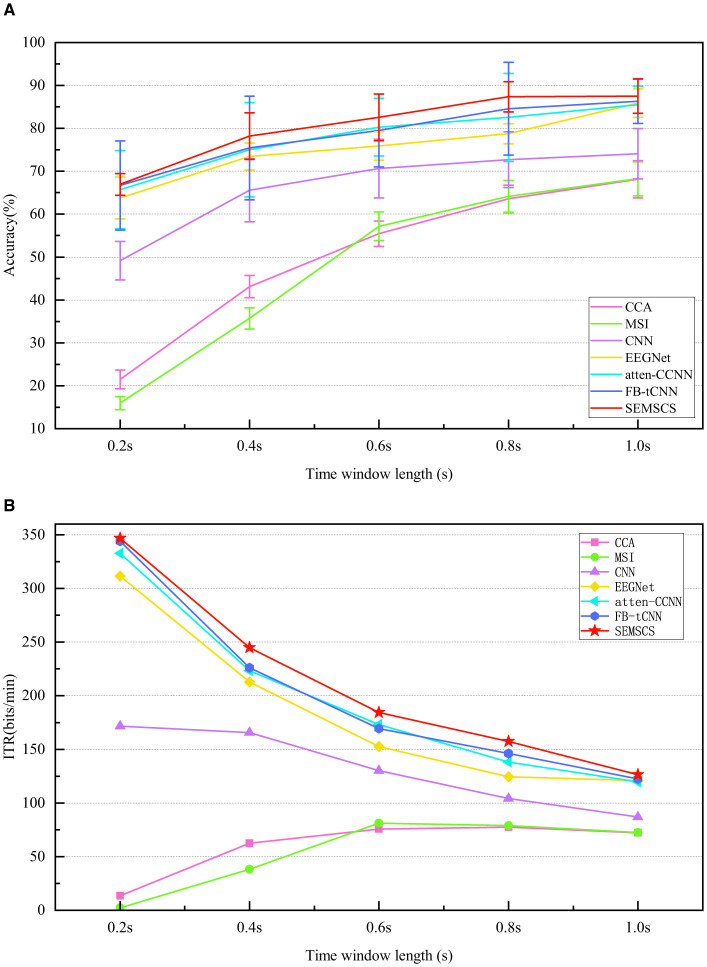
Comparison of performance across seven methods under different time length(s). **(A)** The average accuracy of the seven methods at various time length(s). **(B)** The ITR for the different methods across different time windows.

For all the data, we first conducted within-subject experiments. We calculated the average accuracy and ITR for all subjects using seven different methods with varying data lengths. All data were processed using a non-overlapping sliding window, where the time window was set to [0.5 + *l* + *n*] (Ravi et al., 2020), with 0.5 representing the stimulus onset time, *l* representing five different time window sizes, and *n* denoting the fixed step size for the non-overlapping window. The classification accuracy and ITR obtained by the seven decoding models are illustrated in Figure 12, the detailed data can be found in Table 1.

**Table 1:** Within-subject experimental results: accuracy ± yEr (%) and ITR (bit/min) are presented.

**Data length**	**Index**	**CCA**	**MSI**	**CNN**	**EEGNet**	**Atten-CCNN**	**FB-tCNN**	**SEMSCS**
0.2s	Acc ± yEr	21.5 ± 2.2	16.0 ± 1.5	49.1 ± 4.5	63.8 ± 4.9	65.7 ± 9.1	66.7 ± 10.4	66.9 ± 2.5
	ITR	13.5	2.2	171.7	311.5	332.8	344.1	346.8
0.4s	Acc ± yEr	43.1 ± 2.6	35.7 ± 2.5	65.6 ± 7.3	73.4 ± 3.2	75.0 ± 11.0	75.4 ± 12.1	78.2 ± 5.4
	ITR	62.6	38.2	165.7	212.9	223.3	226.0	244.8
0.6s	Acc ± yEr	55.5 ± 3.0	57.2 ± 3.4	70.6 ± 6.8	75.9 ± 3.3	80.3 ± 6.7	79.5 ± 8.5	82.6 ± 5.4
	ITR	75.8	81.3	130.2	152.7	173.1	169.4	184.4
0.8s	Acc ± yEr	63.6 ± 3.2	64.1 ± 3.7	72.7 ± 6.5	78.8 ± 2.3	82.6 ± 10.3	84.6 ± 10.8	87.4 ± 3.5
	ITR	77.4	78.9	104.1	124.3	138.3	146.0	157.3
1.0s	Acc ± yEr	68.1 ± 4.3	68.2 ± 4.0	74.1 ± 5.9	85.9 ± 3.3	85.5 ± 4.3	86.4 ± 5.2	87.5 ± 4.0
	ITR	72.1	72.4	86.8	120.9	119.7	122.5	126.4

Firstly, traditional methods (CCA and MSI) perform significantly worse across all time windows. Particularly in the shortest 0.2-second time window, the classification accuracy of CCA and MSI is only 21.5% ± 2.2% and 16.0% ± 1.5%, respectively, with ITR values of 13.5 bit/min and 2.2 bit/min, which are far lower than those of the deep learning models. This indicates that traditional methods have limited capacity for processing short time windows, struggling to effectively extract key information from EEG signals, particularly in tasks with high real-time demands. As the time window increases, the performance of traditional methods improves, but it still does not reach the level of deep learning models.

Among deep learning models, CNN and EEGNet outperform traditional methods in short time windows, especially EEGNet, which achieves an accuracy of 63.8% ± 4.9% and an ITR of 311.5 bit/min at 0.2 seconds, showing significant improvement over CCA and MSI. However, these models still do not perform as well in short time windows as they do in longer ones, and their ability to capture temporal features is relatively limited (lower than atten-CCNN, FB-tCNN, and SEMSCS). This may be due to their network architectures, which use a single-layer approach for temporal feature extraction and lack specific mechanisms for channel feature extraction. As a result, these models are unable to fully capture the detailed temporal and channel-specific information from complex signals, which limits their performance. In contrast, atten-CCNN and FB-tCNN show more stable performance in the 0.4-second and 0.6-second time windows, with accuracy above 75% and higher ITR. This improvement is likely due to the more complex feature extraction mechanisms employed by these models, particularly in capturing both temporal and channel features. Unlike CNN and EEGNet, which use single-layer temporal feature extraction, atten-CCNN and FB-tCNN leverage multi-layer, more detailed feature learning techniques (e.g., attention mechanisms and filter banks) that better adapt to complex signals, thereby enhancing their classification performance and information transfer efficiency. However, the more intricate architecture of atten-CCNN and FB-tCNN also results in increased errors and reduced robustness compared to the first two models.

The SEMSCS model demonstrates consistently favorable performance across all time windows, achieving reliable results. In the 0.2-second time window, SEMSCS achieves an accuracy of 66.9% ± 2.5% and an ITR of 346.8 bit/min, significantly outperforming other models. As the time window increases, SEMSCS continues to maintain robust performance, particularly in the 1.0-second window, where accuracy reaches 87.5% ± 4.0% and ITR is 126.4 bit/min. In addition, the SEMSCS model does not experience significant fluctuations in error despite the enhancements in its architecture. The core advantage of SEMSCS lies in its multi-scale convolution and channel feature extraction mechanisms, which effectively capture information across multiple time scales. This approach allows the model to show strong robustness and high classification accuracy, especially in handling long time series. While other deep learning models such as EEGNet, atten-CCNN, and FB-tCNN also perform well under certain conditions, they are less effective when the number of channels is limited.

### 3.3 Cross-subject experiment

Based on the analysis of within-subject experimental results, we observed that all models achieved satisfactory classification performance with a time window of 1.0 seconds. Furthermore, deep learning models outperform traditional methods (CCA, MSI) in terms of classification performance. Therefore, this section focuses exclusively on cross-subject experiments and analysis of five deep learning methods: CNN, EEGNet, atten-CCNN, FB-tCNN, and SEMSCS. Table 2 presents the classification results for different methods across various subjects. The following sections provide a detailed analysis of the cross-subject performance of these methods.

**Table 2:** Comparison of methods on different subjects.

**Subject**	**CNN**	**EEGNet**	**Atten-CCNN**	**FB-tCNN**	**SEMSCS**
S01	75.8%	80.8%	58.6%	80.6%	80.3%
S02	75.4%	80.3%	74.4%	79.2%	82.1%
S03	64.3%	75.4%	70.2%	79.3%	80.2%
S04	58.9%	78.1%	77.0%	74.9%	80.5%
S05	58.4%	82.4%	69.8%	82.6%	84.0%
S06	58.3%	73.6%	67.3%	76.6%	79.2%
S07	71.3%	76.7%	57.2%	78.3%	81.3%
S08	77.0%	82.7%	76.6%	88.8%	84.2%
S09	55.6%	73.6%	73.1%	73.4%	78.4%
S10	70.9%	76.6%	68.4%	79.1%	81.9%
Average	66.6%	78.0%	69.3%	79.3%	81.2%
K-score	0.6181	0.7489	0.6488	0.7632	0.7851
F1-score	0.6564	0.7684	0.6790	0.7804	0.8001
Precision	0.6835	0.7827	0.7077	0.7973	0.8080

In cross-subject tasks, accuracy serves as a direct indicator of model classification performance. Although all models achieve accuracy rates above 60%, SEMSCS outperforms the others with an accuracy of 81.2%. In contrast, CNN shows a significantly lower accuracy of 66.6%, and atten-CCNN performs worse than other deep learning models (EEGNet: 78.0%, FB-tCNN: 79.3%), with an accuracy of 69.3%. Additionally, the kappa score, which reflects model prediction consistency and error levels, further supports these findings. SEMSCS achieves the highest kappa score of 0.7851, indicating its strong consistency and reliable classification results in cross-subject experiments. In comparison, CNN and atten-CCNN have kappa scores of 0.6181 and 0.6488, respectively, indicating poorer consistency across subjects, which aligns with their lower accuracy. FB-tCNN and EEGNet also perform well in terms of kappa scores (0.7632 and 0.7489, respectively), suggesting higher classification consistency. F1-score and precision, as comprehensive evaluation metrics, reveal a similar trend in identifying positive class samples. SEMSCS achieves an F1-score of 0.8001 and a precision of 0.8080, demonstrating high accuracy in recognizing positive class samples while effectively reducing false positives. In comparison, FB-tCNN's F1-score is 0.7804 and precision is 0.7973, slightly lower than SEMSCS but still excellent. EEGNet and atten-CCNN perform worse than FB-tCNN in both F1-score and precision, with CNN showing the weakest performance, with an F1-score of 0.6564 and precision of 0.6835, indicating poor positive class recognition and a higher false positive rate.

These differences may be closely related to the architectural characteristics of each model. SEMSCS integrates an enhanced SE attention mechanism, multi-scale convolution blocks, and deep convolutions from EEGNet, enabling it to effectively focus on key channels, temporal features, and spatial information, thus demonstrating strong adaptability in cross-subject tasks. In contrast, the relatively simple structure of CNN lacks the ability to thoroughly process multi-channel and temporal features, leading to poorer performance in cross-subject experiments. While atten-CCNN introduces an attention mechanism that theoretically enhances feature selection, its traditional convolution layer design fails to adequately capture the diversity of EEG signals across individuals in both the time and frequency domains, limiting its generalization ability. FB-tCNN performs better than atten-CCNN but still falls short of SEMSCS, potentially due to its use of filter banks to suppress interference outside the stimulus frequency range. While this enhances signal clarity to some extent, it may also result in the loss of subtle features, affecting its performance in cross-subject experiments. In summary, SEMSCS stands out with its combination of multi-scale convolution and the SE attention mechanism, demonstrating superior generalization and consistency in cross-subject experiments.

### 3.4 Ablation experiment

To investigate the effect of each module in the proposed network, the SEMSCS network was selected as the baseline model for ablation studies. Cross-subject training was conducted using data with a time length of 1 second from 10 subjects. The decoding performance of the ablation experiments is presented in Table 3. The first row shows the decoding performance of the baseline model, while the subsequent rows (rows 2–5) display the results obtained by progressively removing each of the four blocks. Furthermore, to further evaluate the effectiveness of the improved SE attention mechanism, we introduced traditional SE, SK, and self-attention mechanisms while retaining the Block 2-3 structure in the network. The experimental results are shown in rows 6–8 of Table 3.

**Table 3:** Results of ablation experiment.

**No**.	**Block 1**	**Block 2**	**Block 3**	**Block 4**	**SE**	**SK**	**SA**	**Acc**	**K-score**	**F1-score**	**Precision**
1	✓	✓	✓	✓				81.2%	0.7851	0.8001	0.8080
2		✓	✓	✓				78.9%	0.7590	0.7739	0.7940
3	✓		✓	✓				75.9%	0.7250	0.7443	0.7691
4	✓	✓		✓				78.4%	0.7535	0.7736	0.7936
5	✓	✓	✓					73.8%	0.7004	0.7267	0.7438
6		✓	✓	✓	✓			79.5%	0.7662	0.7813	0.7978
7		✓	✓	✓		✓		78.6%	0.7558	0.7730	0.7889
8		✓	✓	✓			✓	79.3%	0.7634	0.7784	0.7936

Using the baseline model as a reference, comparisons were made with rows 6, 7, and 8. In row 2, removing Block 1 (the improved SE attention mechanism) led to a decrease in accuracy to 78.9%, with other metrics also showing a downward trend. This 2.3% reduction from the baseline model (81.2%) suggests that Block 1, as the improved SE module, plays a role in extracting features from non-characteristic channel data under limited electrode conditions. Replacing Block 1 with the original SE module (row 6) increased accuracy to 79.5%, indicating that the original SE module can recover some performance in channel attention modeling, though it still underperforms compared to the improved SE module. In contrast, replacing Block 1 with the SK attention mechanism (row 7) resulted in a further decrease in accuracy to 78.6%, slightly lower than removing Block 1 (78.9%). While the SK attention mechanism dynamically selects features across different scales, its main role is feature fusion, which may not effectively extract features from non-characteristic channel data with limited electrodes. Replacing Block 1 with the self-attention mechanism (row 8) resulted in a slight recovery, with accuracy increasing to 79.3%, higher than deleting Block 1. This suggests that self-attention enhances inter-channel information exchange through global feature modeling, but its performance remains lower than the improved SE module, possibly due to less precise capture of local non-characteristic channel information during global attention weighting.

In the experiments removing Block 2 to Block 4, the contribution of each module was further confirmed. Removing Block 2 (row 3) significantly decreased accuracy to 75.9%, emphasizing the importance of Block 2 in multi-scale temporal feature extraction. In contrast, removing Block 3 (row 4) resulted in a smaller decrease, with accuracy remaining at 78.4%, suggesting a more moderate effect of Block 3's feature fusion on overall performance. The most significant drop occurred when Block 4 was removed (row 5), with accuracy falling to 73.8% and a notable decrease in F1 score. This indicates the critical role of Block 4 in extracting spatial features, especially when the number of channels is limited, as the loss of spatial information led to substantial performance degradation.

### 3.5 ROS online simulation

**Figure 13:**
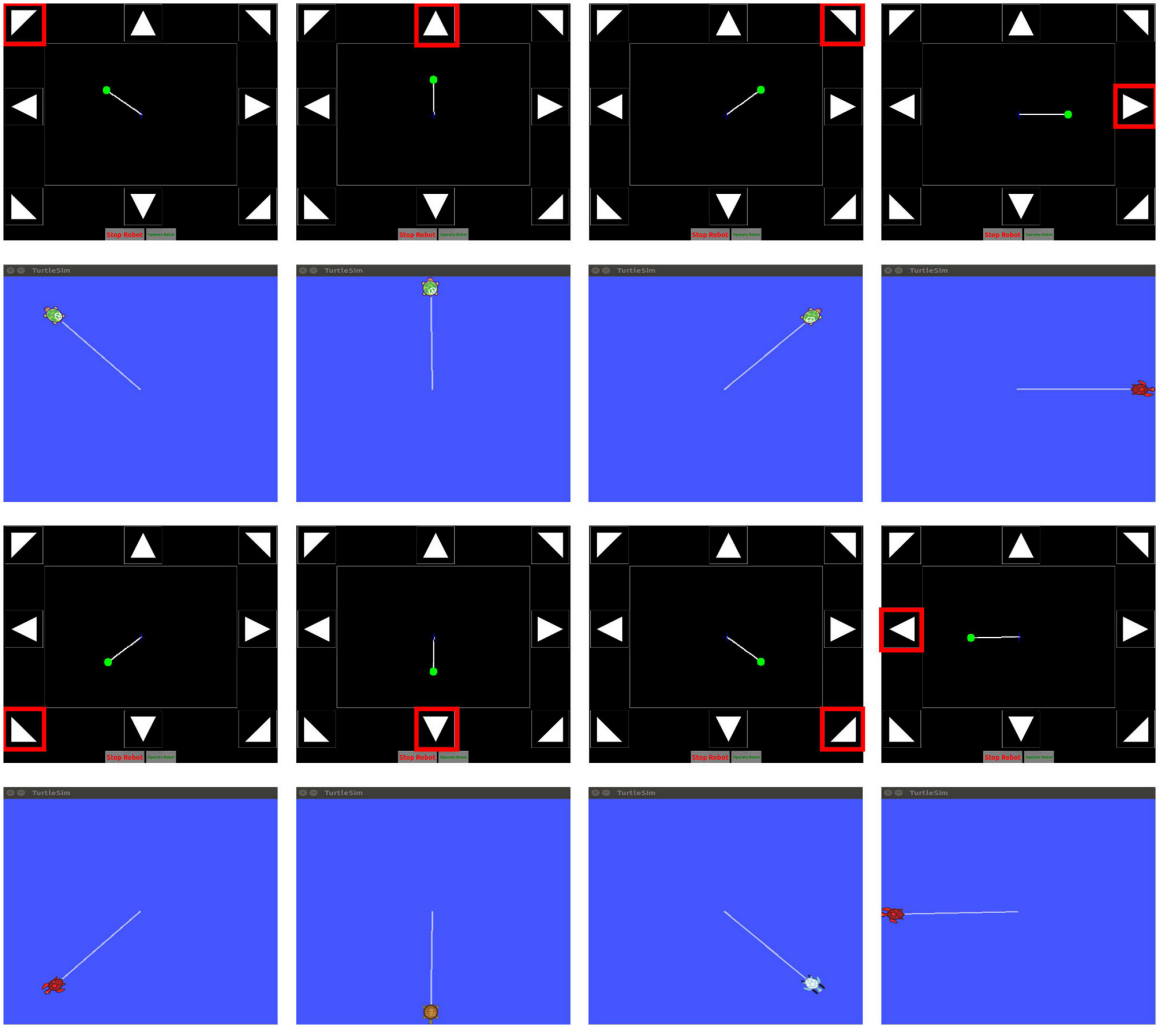
Online simulation validation using the ROS turtlesim simulator and the gaitech_bci_teleop package.

Online experiments were conducted using the Gaitech BCI and SEMSCS during the night, when fewer unrelated personnel were present, with external interference minimized by turning off unnecessary devices and maintaining a stable light source to reduce the impact of 50 Hz electrical noise. A turtlesim simulator was created in the ROS environment, allowing the turtle's position, orientation, and other information to be accessed via topic messages for control. Package 1 sends the collected data to Package 2, where real-time filtering is performed. The filtered data is then published to the model. ROS Bridge enables data transmission across different environments and languages. The model outputs the classification label, which is then published to the script in Package 3. The Python script was used to publish messages to the “cmd_vel” topic, a standard ROS topic for robot control. The script included eight commands corresponding to the eight SSVEP stimuli, with each command defining a fixed movement direction for the turtle, while the speed remained constant. An “image_topic” was implemented to receive the turtle's pose, which was displayed in “gaitech_bci_teleop” to visualize the turtle's movement trajectory. This setup facilitated comparison with the path generated in the simulator, enabling online validation of the SEMSCS model's performance in controlling robot motion through SSVEP signals. As shown in Figure 13, the figure presents the turtle's movement controlled by the eight target classes, alongside the pose images returned by the “image_topic.”

## 4 Conclusion and discussion

In this study, we developed a data acquisition and analysis framework for portable wearable BCI devices based on the ROS system and proposed the SEMSCS model, which incorporates an improved SE attention mechanism. The model effectively utilizes limited electrode data and enhances feature selection, particularly in scenarios with restricted channel data. The Gaitech BCI system demonstrated practical utility, offering a reliable solution for EEG signal acquisition and analysis, as well as a feasible approach for controlling external devices. Environmental factors, such as power line interference and external noise, can affect signal quality. In this study, we used Gaitech BCI's Package 2, which includes high-pass, low-pass, and notch filters, along with measures like turning off unnecessary devices and using stable light sources, to minimize external noise and ensure data reliability. However, physiological noise, particularly due to individual differences among participants, remains a challenge. Despite substantial research on suppressing eye, muscle, and heart artifacts, the complexity of individual variations requires further exploration. Future work will focus on increasing the participant pool to collect more diverse data, analyzing individual differences, and developing personalized noise suppression techniques. Additionally, we plan to improve the experimental setup by simulating more dynamic, real-world environments to enhance the system's reliability in practical applications.

While the SEMSCS model showed good performance with the current dataset, the small sample size may lead to overfitting. Expanding the dataset will not only address this concern but also help validate the model's generalizability. Future research will explore methods to reduce overfitting and optimize computational efficiency, especially in resource-constrained environments, as model complexity increases.

With the increasing adoption of portable BCI devices, our work may open new avenues for their application in healthcare, education, and assistive technologies. The SEMSCS model, combined with portable EEG devices, could offer more flexible and efficient brain-machine interfaces for clinical patients, supporting motor rehabilitation and health monitoring. Additionally, the ROS-based system may facilitate easier integration with external devices, enhancing scalability for educational settings and improving EEG signal analysis. Continued optimization could further advance the deployment of BCI technologies across various fields.

## Data Availability

The datasets presented in this article are not readily available because they are subject to ethical and privacy restrictions. Requests to access the datasets should be directed to the corresponding author.
